# Variation in the circularly polarized light reflection of *Lomaptera* (Scarabaeidae) beetles

**DOI:** 10.1098/rsif.2016.0015

**Published:** 2016-07

**Authors:** I. E. Carter, K. Weir, M. W. McCall, A. R. Parker

**Affiliations:** 1 The Blackett Laboratory, Imperial College London, Prince Consort Road, London SW7 2AZ, UK; 2 Department of Life Sciences, Natural History Museum, Cromwell Road, London SW7 5BD, UK

**Keywords:** scarab beetle, circular polarization, structural colour, chiral, birefringent, chitin

## Abstract

An extended spectroscopic study on the left-through-left circularly polarized reflection spectra of a large number of beetles from the Australasian Scrabaeidae:Cetoniinae of the *Lomaptera* genus was undertaken. We have obtained a five-category spectral classification. The principal spectral features, which even within the genus range from blue to infrared, are related to structural chirality in the beetle shells. The detailed features of each spectral classification are related to different structural perturbations of the helix, including various pitch values and abrupt twist defects. These spectral characteristics and associated shell structures are confirmed on the basis of simple modelling. An important conclusion from our study is that the simple helical structure resulting in a single symmetric Bragg peak is not the dominant spectral type. Rather the reality is a rich tapestry of spectral types. One intriguing specimen is identified via a scanning electron micrograph to consist of a double interstitial helix leading to a particular double-peak spectrum.

## 1. Introduction

For over 100 years since Michelson's discovery of the circularly polarized (CP) light mechanism responsible for iridescence in Scarabaeidae beetles [1], this has been a subject of study across a wide range of fields, including biology, optics and even engineering [2]. In 1924, Gaubert observed that these beetles appeared to reflect left circularly polarized (LCP) light [3]. It was later shown by Neville & Caveney [4] that a small minority of beetles reflected right circularly polarized (RCP) light. This prompts further interest as there are very few species in Nature which interact with CP light that include the marine stomatopod *Odontodactylus* and several species of firefly [5–8].

A general study on the reflection from a wide range of different beetles of the degree of CP light and colour was conducted by Pye, who found optically active (induces change in the polarization of incident light upon reflection) species in the Scarabaeidae and Hybosoridae families, which mainly reflected LCP light [9]. The advantages of this selective reflection are as yet not fully understood. It is however known that several species of Scarabaeidae can navigate using Rayleigh scattered (or polarized) light from the moon [10].

## 2. The optical response of the beetle cuticle

**Figure 1.**
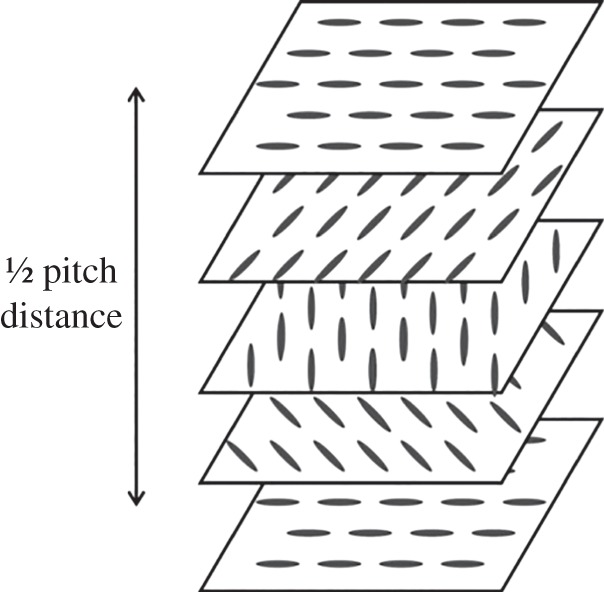
Schematic of a liquid crystal in the cholesteric phase displaying the gradual change in orientation of the thread-like molecules over consecutive layers of the material.

The beetles examined in this study have a very distinctive appearance. Typically, they are green (though other visible wavelengths have been observed) and they have an iridescent, metallic sheen. As previously mentioned, earlier studies have noted the distinctive polarization characteristics of the reflected light and, together with microscope examination of the structure of the beetle's carapace, this has resulted in a simple model of the reflection mechanism. The beetle's shell is taken to primarily consist of thin layers of thread-like molecules (chitin) with all the threads aligned parallel within a single layer. The anisotropy of the molecules means that the layers are birefringent. As these layers build on top of each other, there is a small angular change between consecutive layers which gives rise to a helical structure through the depth of the shell. The birefringence is an important feature of the individual layers, as it contributes to the ‘strength’ of the helix; indeed, the uric acid in the shell enhances the birefringence of the layers [11]. Once there are enough layers to form a full 360° rotation, the pitch of the helix is defined. This helical structure is similar to the cholesteric liquid crystal phase as illustrated in figure 1. A typical beetle shell will be thick enough to consist of tens of pitches (though not necessarily an integer number). The reflection characteristic of the structure is then determined by the Bragg resonance of incident light and the helical structure—the wavelength at which there is maximum reflectance being determined by the pitch of the helix and the polarization state by the handedness. This wavelength, *λ_p_*, at which there is a maximum reflectance is given by2.1
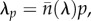
where 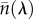 is the average refractive index of chitin [12] and *p* is the structural pitch. This is analogous to the (scalar) response of Bragg gratings [13,14].

The circular polarization induced metallic colour is less dependent on viewing angle than linearly polarized induced colour [15]; however, it has been observed that at some angles this effect is reduced [16]. The reflection of the CP light from the beetles preserves handedness and does not induce a phase shift [17]. Several variations from a single-pitch structure have previously been seen. One interesting observed variation is a half-waveplate-like layer in the middle of the helix of a *Chrysina resplendens* beetle [11], resulting in the beetle reflecting both LCP and RCP light. Electron micrographs of this species taken by Neville [18] confirm the broadband spectral features of this structure. A double-pitched structure in a *Chrysina boucardi* beetle has also been seen using a transmission electron microscope [15].

The majority of previous experimental investigations of beetle cuticles have considered the wavelength of peak reflection (or general ‘colour’ [9]) and its polarization state. This study focused upon measuring the visible spectra of LCP light reflected from the cuticle of beetles of the *Lomaptera* genus. The spectral characteristics were used to consider variations on the simple structure outlined above and as a potential tool for characterizing species within this family.

## 3. The beetles studied and the experimental approach

The Natural History Museum, London, has an extensive collection of beetles (almost 10 million specimens). Initial investigations sought to identify a family of beetles which gave rise to a strong polarization response. Consequently, in this study, the LCP reflection spectra of 209 beetles drawn from 38 species of *Lomaptera* beetles (Coleoptera: Scarabaeidae: Centoniinae) were studied in detail. These specimens strongly reflected CP light and provided an acceptable signal-to-noise ratio across the spectra (it should be noted within this family that 19 other *Lomaptera* species showed weak CP response, with 28 showing no CP light response).

**Figure 2.**
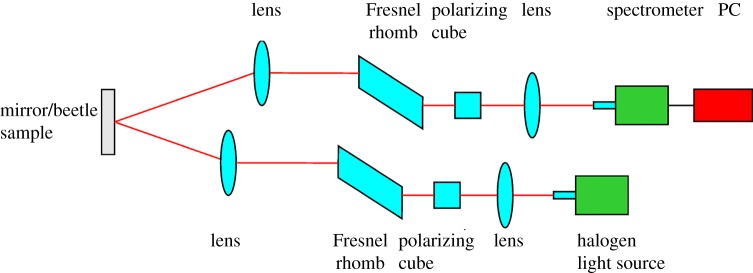
Experimental set-up in order to measure the CP response from the *Lomaptera* beetles. (Online version in colour.)

A schematic of the experimental arrangement is shown in figure 2. The light from an Ocean Optics DH2000 BAL halogen light source (400–1200 nm) [19] was transmitted by an optical fibre and the collimated output from the fibre (Ocean Optics QP600-2-SR-BX) was polarized to be LCP. The LCP light was generated by passing the unpolarized light through a polarizing cube, which is set to produce linearly polarized light at +45° to the axis of the Fresnel rhomb. Inside the Fresnel rhomb two internal reflections occur, producing a total phase shift of *π*/2 resulting in LCP light [20]. This approach minimizes the wavelength dependence in producing LCP light. This light was then focused to an even 1 mm diameter spot incident upon the scutellum (an approximately flat, triangular-shaped plate) of the beetle's thorax (or mirror for the calibration). The reflected light was collected by an optical arrangement similar to the input stage with the Fresnel rhomb and polarizing cube orientated to allow the transmission of LCP light. The transmitted light was coupled into a fibre which was then input to an Ocean Optics HR4000 spectrometer [21], which recorded the spectrum between 450 and 1000 nm. The recorded spectra were passed to a computer for analysis. The system was set to have 45° between the input and output arms in order to be close to normal incidence while allowing space for the optical arrangement.

If required, the orientation of the polarizing cubes in the input and output arms could be rotated through *π*/2 to provide incident RCP and record RCP reflected light. Here, it is the LCP response that is examined as this provides the major response and contains the most information; in general, the opposite handedness provided a weak, featureless response.

Before taking any measurement on a beetle, a mirror is used as a reference in order that the reflectivity takes into account the spectrum of the halogen light source. Thus, once the reflection spectra were recorded from a beetle (*signal*(*beetle*)), the reflectance could be calculated via3.1
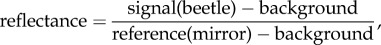
where ‘background’ represents the dark response of the spectrometer and was recorded with no light input from the light source.

The LCP reflection data are discussed in detail in the following section and present new distinctive features compared with the simple response discussed previously. To provide a framework for the discussion of the key features of the spectra and their possible structural origins (how they vary from the basic single-pitch structure), the shapes of the spectral features were grouped into five categories: (i) single peak, (ii) double peaks close together, (iii) double peaks with distinct peaks, (iv) diminishing oscillations before the main peak, and (v) diminishing oscillations after the main peak. Reflection spectra that did not clearly fit into one of these categories were described as ‘not classified’.

The observed spectra are compared with spectra modelled on an assumed structure for the beetle's shell. The model is implemented as a multilayer transfer matrix method using the Birefringent Thin Films Toolbox described in [22]. It should be noted that all the models are not a perfect fit and are used to demonstrate how small changes in the cuticle structure can result in different optical spectra. There was slight variation of spectral shape and wavelength within the five categories; as such the model would need to be adjusted for every spectra. The modelling was done with several different models: a simple single pitch, a defect in the rotation, a chirped structure, absorption and also multiple pitch values. The refractive index taken to model chitin was within the given range *n* = 1.4–1.8 [23], and the birefringence can vary between *Δn* = 0.018 and 0.084 [24], depending upon the chemical composition of the helical structure, and is often enhanced by uric acid. The thickness of the chitin layers used was *d* = 20 nm and was also taken from the literature values [25]. Further detailed discussion of how the simulations alter changing the number of layers and using various structural defects can be found in the electronic supplementary material.

## 4. Left circularly polarized spectral types

### 4.1. Single peak

The single-peaked spectrum is the response anticipated for the simple model outlined previously.

**Figure 3.**
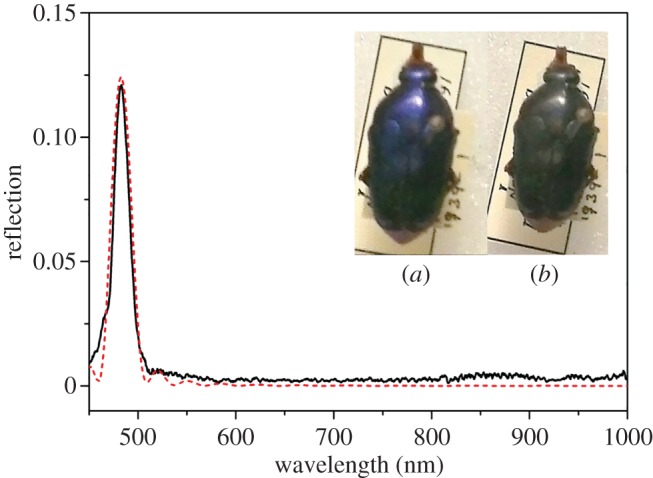
Single-peak LCP reflection graph with a clear single peak from a *L. pygmaea* specimen (black curve). The insets show the appearance of the specimen through (*a*) left and (*b*) right circular polarizers. The red-dashed plot shows a theoretical plot of the left-through-left reflectivity with 10 pitches thick with each individual chitin layer of 20 nm and birefringent refractive indices of 1.475 + 0.008i and 1.50 + 0.008i.

**Figure 4.**
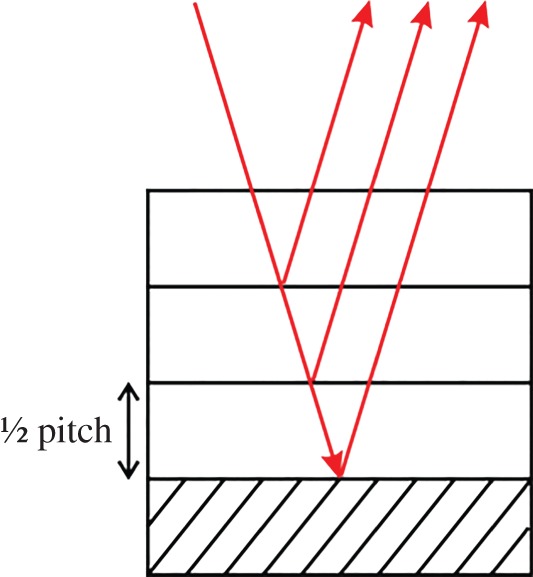
Schematic of a single-pitch stack which gives rise to a single-peak spectrum. (Online version in colour.)

Figure 3 shows an example of the least complex of the experimentally observed responses, being a result of a reflection from a regular stack of chitin layers forming a single uniform pitch. The wavelength corresponding to the maximum reflectance is related to the pitch as in equation (2.1). The model for the structure in this situation is the single-helical structure illustrated schematically in figure 4. The theoretical response, modelled on a simple single-pitch structure, is shown in figure 3 (the red-dashed response). This spectral type was observed for 29 of the 209 beetles (13.9%) that had their spectra recorded and analysed, though the peak wavelength did vary between species. This variation in peak wavelength is another aspect of the characterization which will be discussed further.

### 4.2. Double peaks

**Figure 5.**
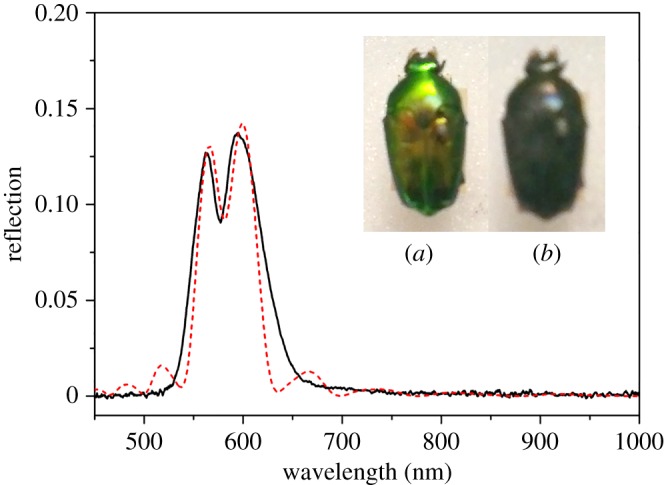
Double-peak LCP reflection graph with a small trough from a beetle of the species *L. pygmeae* (black curve). The insets show the appearance of the specimen through (*a*) left and (*b*) right circular polarizers. The red-dashed theoretical curve is modelled upon a discontinuity in the pitch of 8/15*π*, using complex birefringent refractive indices of 1.46 + 0.025i and 1.52 + 0.025i to simulate absorption, and a layer 11 pitches thick in total.

Other easily classified shapes of LCP reflection spectra were those that included double peaks. There are two distinct types: first, double peaks which are close together and can also be considered as a single peak with a narrow trough. An example of this type of spectrum is shown in figure 5 for a *L. pygmaea* beetle. It is to be noted that this spectral characteristic cannot be distinguished from the image of the beetle (figure 5*a*). A simple theoretical model based upon a sudden jump of (8/15)*π* in the orientation of the chitin molecules within a single layer was used to generate a theoretical spectrum (the red-dashed response shown in figure 5). The value chosen for the orientation jump was to optimize the asymmetry between the peak heights.

This spectral type was observed for 46 of the 209 beetles (22.0%) that had their spectra recorded and analysed, though the peak wavelengths did vary between species.

**Figure 6.**
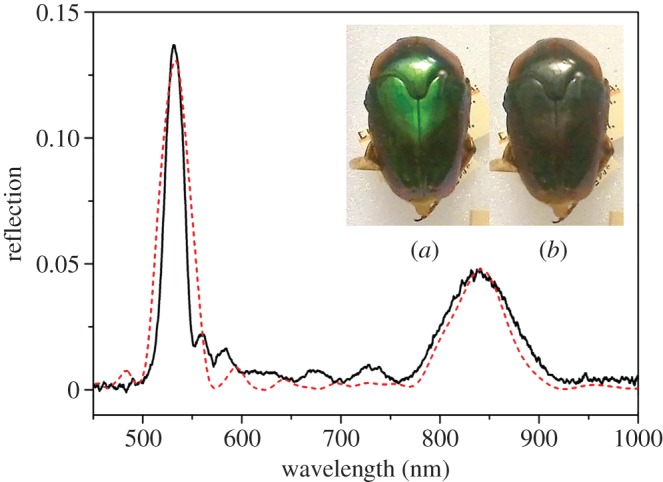
Double-peak LCP reflection graph with two clearly distinct peaks from a *L. geelvinkiana* specimen (black curve). The inset photos show the appearance of the specimen through (*a*) left and (*b*) right circular polarizer. The red-dashed theoretical curve is modelled upon two distinct pitch values, using complex birefringent refractive indices of 1.485 + 0.01i and 1.52 + 0.01i and having a thickness of 12.5 pitches in total.

The second classification of double peaks differs in that the peaks appear as two clearly separate, distinct peaks as illustrated in figure 6. This was theoretically modelled as two distinct pitches, with the lower wavelength closer to the surface using birefringent refractive indices of 1.46 and 1.52. This response is shown in figure 6 (the red-dashed response). Clear oscillations between the two main peaks are features which are observed both in the experiment and theoretical models.

This spectral type was observed for eight of the 209 beetles (3.8%) that had their spectra recorded and analysed, though the peak wavelengths did vary between species.

**Figure 7.**
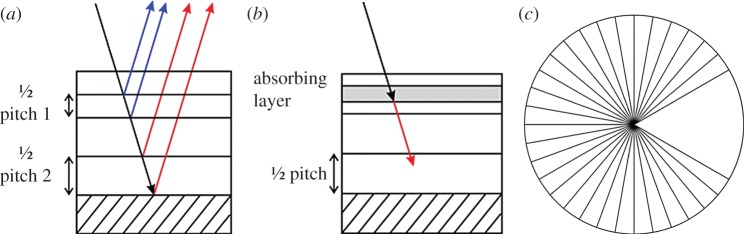
Three different structures, which could cause double-peak structures in multi-layered structures: (*a*) a structure of two distinct pitches, (*b*) the structure includes a layer which absorbs specific wavelengths or wavebands and (*c*) a chiral structure with a twist defect (a step in the orientation of the chitin molecules between adjacent layers). (Online version in colour.)

**Figure 8.**
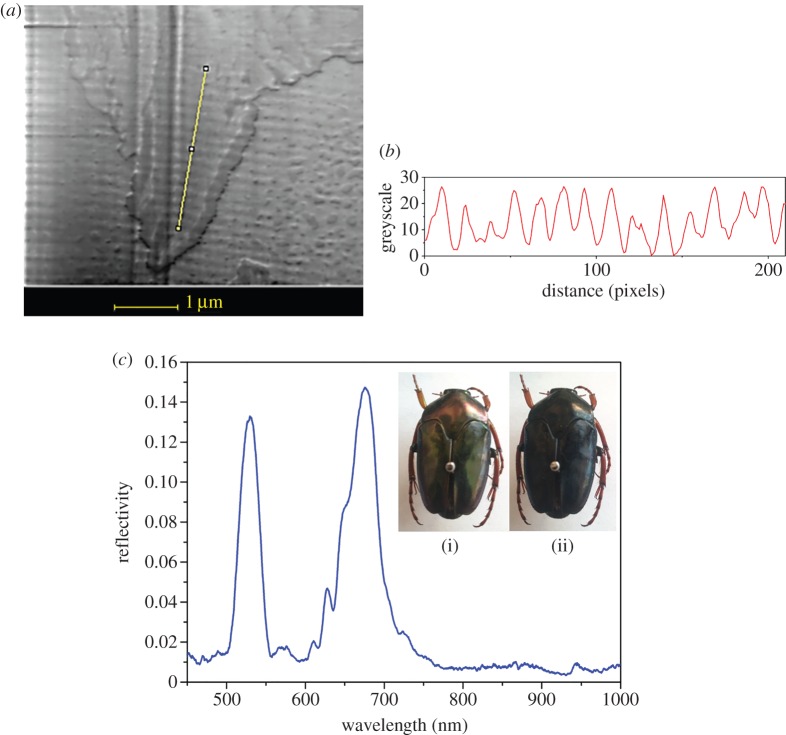
(*a*) Electron microscope image and cross-sectional image of the beetle *Mycterophallus validipes*' scutellum. (*b*) A greyscale profile taken along the line on the image in (*a*). (*c*) The associated optical spectrum of LCP light with image of beetle through (i) LCP filter and (ii) RCP filter. (Online version in colour.)

There are three variations on the simple chiral structure of the beetle shell which could give rise to the two different kinds of double-peak spectra (figure 7): (a) two distinct pitch values, (b) an absorbing layer within the structure and (c) a sudden step in the orientation of the chitin molecules between adjacent layers which disrupts the continuous helix. An absorbing layer present among the chitin layers would stop certain wavelengths of light from being reflected, and hence this would cause a trough at these wavelengths, which is more likely to occur in the double peaks which are further apart. The second type being the two distinct pitch values which correspond to two different wavelengths of light which are reflected. Such a structure has been reported by Jewel *et al*. [15]. The third structure is characterized by a sudden change in the chitin helix, known as a twist defect [26]. While the double peaks close together could be caused by either (a) or (c), having compared the relative merits of these models in the supplementary material it is concluded that only (c) is possible. The double peaks further apart are most probably caused by the two distinct pitch values. The double-pitch hypothesis is supported by a scanning electron micrograph of the cross section of the shell of a non-museum *Mycterophallus validipes* beetle (a *Lomaptera* beetle is a closely related and geographically similar non-museum specimen) which shows the distinctive separate peaks. The electron micrograph is shown in figure 8*a,b* and a greyscale profile was taken and plotted. Owing to the low contrast in other parts of the image, only a limited dataset was available for detailed analysis. Therefore, the period was estimated by measuring it directly from the profile. The measured values fell naturally into two groups. The mean wavelength of the optical structures in each of these groups was of 531 and 682 nm, which correspond closely to the values determined from the peak reflection of 528 and 673 nm (figure 8*c*).

The double-peaked spectral types were observed for 54 of the 209 beetles (25.8%) that had their spectra recorded and analysed, though the peak wavelength did vary between species.

### 4.3. Spectra with oscillations

**Figure 9.**
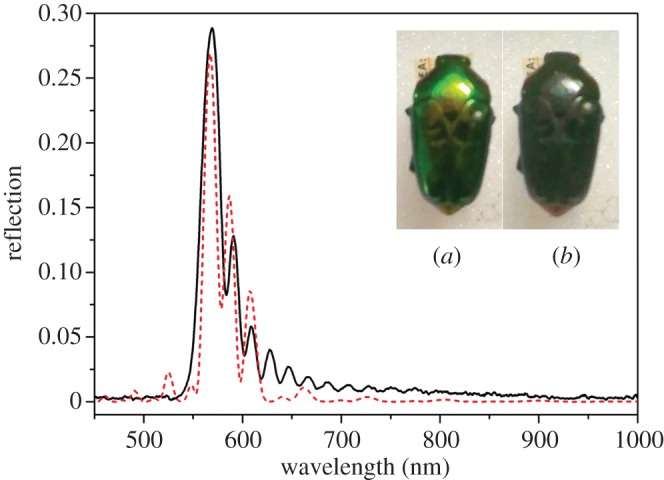
LCP reflectance spectrum with gradually reducing oscillations at higher wavelengths of a beetle of *L. pseudodichropus* (black curve). The insets show the appearance of the specimen through (*a*) left and (*b*) right circular polarizers. The red-dashed theoretical curve is based upon a model of a structure 19.5 pitches thick and discontinuities in the pitch of (1/6)*π* and (1/2)*π*, using complex birefringent refractive index of 1.498 + 0.002i and 1.52 + 0.002i.

**Figure 10.**
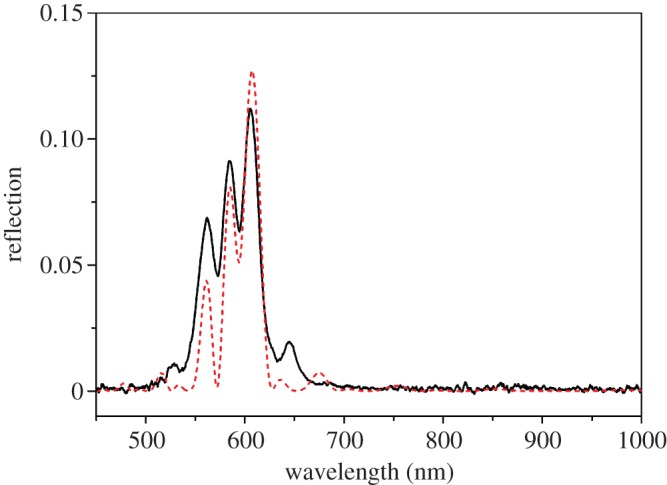
Experimental LCP reflectance spectrum with gradually increasing oscillations before the main peak of a beetle of *L. soror* (black curve). The red-dashed curve is based upon a theoretical model with two different twist defects. The model is based on a structure 16.5 pitches thick with discontinuities in the pitch of (5/6)*π* and (3/2)*π*, using complex birefringent refractive indices of 1.498 + 0.006i and 1.52 + 0.006i.

The third group of characteristic LCP reflection spectra included oscillations around the main peak. There are two distinct types. The first being where there are oscillations that reduce in intensity as they move away from the main peak towards longer wavelengths (figure 9). The second being oscillations that decrease in intensity as they move away from the main peak towards shorter wavelengths (figure 10). Modelling of the oscillations was achieved in a similar manner for both cases. Two discontinuities in the orientation of the chitin molecules were introduced and equally spaced within the structure. For the oscillations after type, the discontinuities were (1/6)*π* and (1/2)*π* (red-dashed response in figure 9). The model for the oscillations before the main peaks introduced two discontinuities of (5/6)*π* and (3/2)*π* (red-dashed response in figure 10).

The ‘oscillations before’ spectral type was observed for eight of the 209 beetles (3.8%) that had their spectra recorded and analysed, and the ‘oscillations after’ for 66 of the 209 beetles (31.6%).

**Figure 11.**
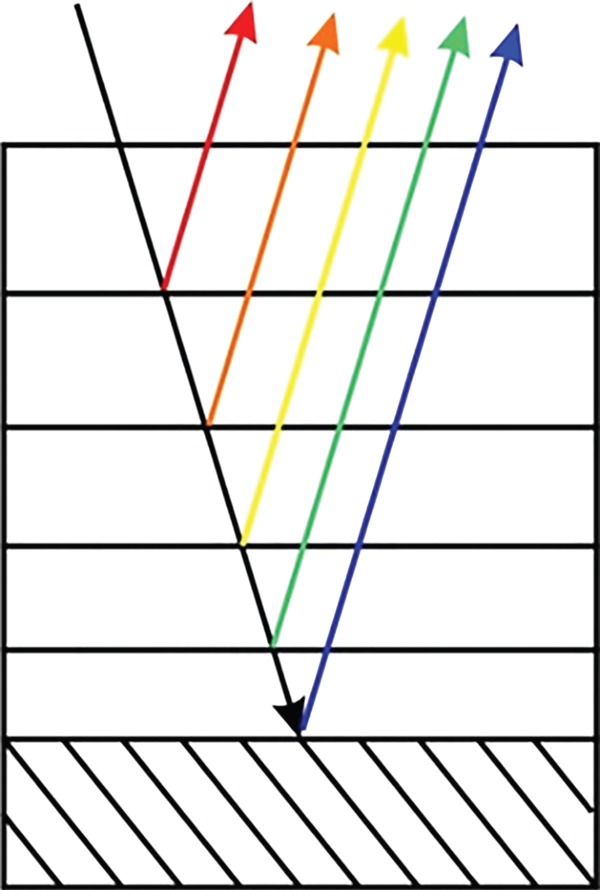
A possible structure behind the production of oscillations in the beetles' reflection spectra. This chirped structure illustrates the situation where the layers decrease in thickness with depth.

The gradually diminishing oscillations were previously described as being caused by chirped structures, with oscillations after the main peak indicative of increasing pitch thickness the deeper into the beetle shell. However, oscillations before the main peak were indicative of the situation where the pitch thickness decreases away from the surface (figure 11). Such a structure has previously been discussed by Parker *et al*. [27].

### 4.4. Other spectra

**Figure 12.**
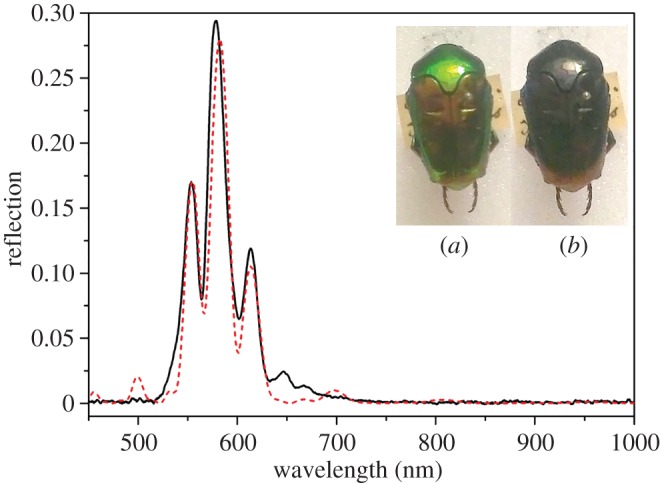
A plot of an LCP spectrum, this being one from a *L. soror* beetle, showing a response which could not be placed into the five chosen classifications (black curve). The red-dashed theoretical curve is from a model based upon a structure 13.5 pitches thick with discontinuities in the pitch of (1/15)*π* and (17/24)*π*, using complex birefringent refractive indices of 1.49 + 0.001i and 1.52 + 0.001i.

Finally, the ‘not classified’ LCP spectra were varied in their features with most not being simple double-peaked or with gradually increasing/decreasing oscillations, but somewhat more complex (figure 12). A model was created based upon two discontinuities, to describe a typical unclassified spectrum, with two discontinuities in the orientation of chitin molecules of (1/24)*π* and (3/4)*π*, along with complex birefringent refractive indices, simulating absorption (red-dashed trace in figure 12).

Fifty-two of the 209 beetles (24.9%) that had their spectra recorded as not classified.

## 5. Spectral analysis and classification

**Figure 13.**
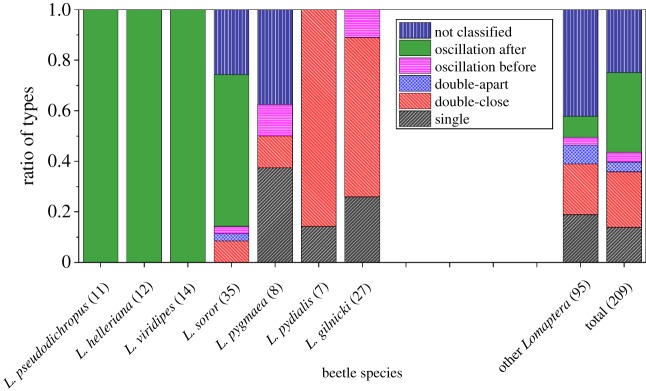
Column chart of the distribution of different shape LL graphs within different beetle species. It should be noted that the diagram does not include species of *Lomaptera* where there were fewer than seven specimens. The number in parentheses after the species name denotes the number of specimens of a species/type which were sampled.

Analysis of the reflection spectra types was done within and between species of the *Lomaptera* genus and the results brought together to show the extent that each spectral type occurred for each species. This is illustrated in figure 13; it can clearly be seen that there are differences between the species and also within them. It should be noted that where the total number of samples for a species was small they were bought together as ‘other *Lomaptera*’.

The data of figure 13 highlight some important distinctions. *Lomaptera pseudodichropus, L. helleriana and L. viridipes* species consistently show oscillations after the main peak, whereas in the *L. gilinicki* species a wider range of spectral shapes occur, none of which are oscillations after the main peak. This result demonstrates that a *L. viridipes* beetle could be distinguished from a *L. gilinicki*, and a simple classification can be made. However, this is not a conclusive method of classification since shapes alone cannot identify a single species, notably a distinction between *L. pseudodichropus, L. helleriana* and *L. viridipes* cannot be made. As well as between these species, there is considerable variation within some species. For instance, *L. soror* shows four basic spectral shapes as well as having some spectra that were not classified.

The frequency of the spectral shape classification across all species is summarized in table 1 and highlights another feature of the typical structures present in beetles' cuticles—the simple single helix structure outlined earlier is *not* the dominant structure. Indeed, it is the spectra which would suggest a range of variation in the fine detail of the structure which dominate.

**Table 1.** The relative frequency of the spectral shapes across all of the *Lomaptera* beetles included in this study.

spectral shape	single peak	double-close	double-apart	oscillations before	oscillations after	not classified
frequency (%)	13.9	22.0	3.8	3.8	31.6	24.9

Another characteristic of the spectra that could be determined and analysed across the specimens is the wavelength of the peak reflectance. The peak wavelength is readily obtained from the spectra and the corresponding pitch of the chiral structure calculated from equation (2.1). The refractive index of the chitin [12] was obtained using the wavelength-dependent Sellmeier relation:5.1
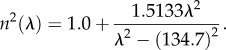


While it is a simple linear relationship between peak wavelength and pitch, it has been chosen to present the following data in terms of pitch as this relates directly to the structure of the beetle cuticle.

**Figure 14.**
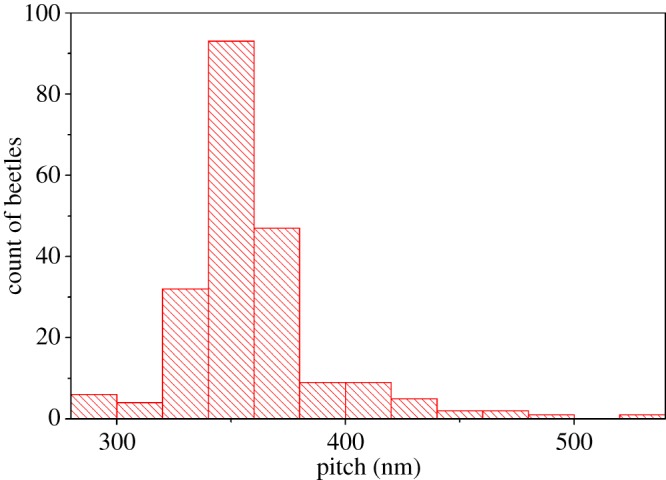
Histogram of all beetle pitch values (nm) from the main peaks of the beetles' LCP reflection spectra, taking into account both pitches in a double-peak spectrum and omitting unclassified spectra. (Online version in colour.)

The pitches of all the *Lomaptera* beetles were obtained and plotted as shown in figure 14. For double-peaked spectra, the pitch values for both peaks were included; the data for not classified spectra were omitted. It can be seen that 81.5% of all *Lomaptera* pitches were between 320 and 380 nm, corresponding to a reflected wavelength that is consistent with the predominant green colour observed. However, there were clear outliers with pitches less than 300 nm and more than 500 nm corresponding to blue wavelengths (450 nm) and near-infrared wavelengths (800 nm) (whose LCP selective reflection could not be observed visually). It should be noted that contrary to previous publications where a black *Lomaptera* beetle had a layer of melanin in front of the optically active structure blocking the structural colour [28], beetles observed as black in this study simply were reflecting beyond the visible range with the black colour coming from a layer of melanin behind the optically active structure.

**Figure 15.**
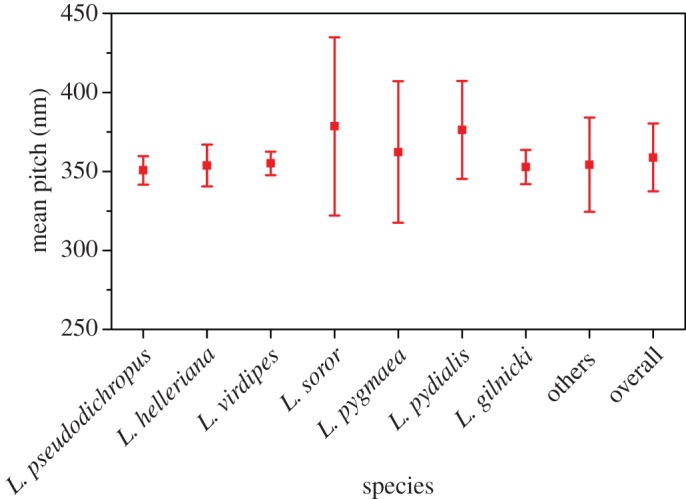
The mean pitch and standard deviation error across species. (Online version in colour.)

There are many ways in which the calculated pitches can be investigated in order to identify characteristics that may be linked to an individual species or a spectral shape. Figure 15 shows the mean pitch for each of the species with the error bars representing the standard deviation. There are clear distinctions between species. The species dominated by a single-peak response have a narrow distribution and the mean pitch between species shows a small variation. Thus, this feature alone is not sufficient for classification purposes.

**Figure 16.**
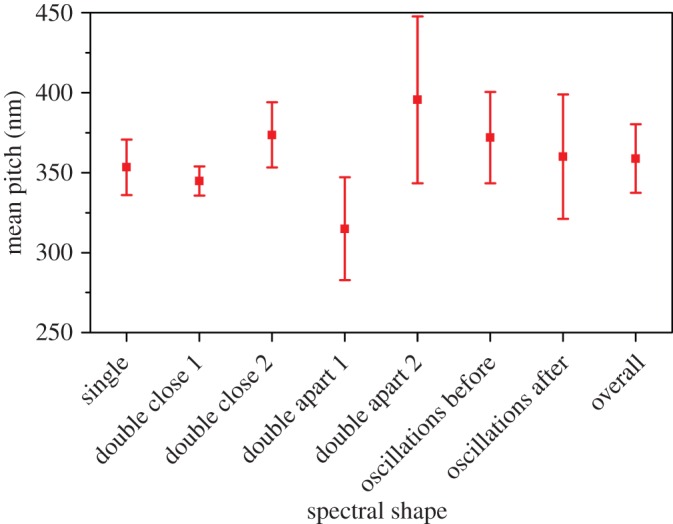
The mean pitch and standard deviation error across different spectral shapes. (Online version in colour.)

Figure 16 shows the mean pitch and standard deviation across spectral types. This reaffirms the narrow distribution of single peaks across all spectral types, the second peak in double peak close together spectra showing a wider variation. By definition, for the double peak far apart spectra the mean pitches are quite distinct, and it is noted that these values lie outside the range defined by the single-standard deviation around the mean pitch of the single-peak spectra. Considering the values of mean pitch and their standard deviation across all species and spectral types in general (and excluding double-peak far apart) the variation of mean values is small at 30 nm which represents a variation in the pitch of less than 10%. This may suggest that the molecular structure of the beetle cuticle is such that it will always be in this range, and the variations in the mean pitch are subtle responses to external factors. The double peak far apart spectra may seem to contradict this. However, if the structure is indeed the intertwined pitches there are not two individual pitches and the structure again reveals subtle changes in the physical dimension of the layers.

## 6. Variations

As discussed, the variations in the LCP reflection spectra arise from subtle changes in the structure; however, the reasons as to why they develop are still not clear. Variations could be caused by a large number of factors. These include those which are related to the growth of the beetle such as the age and date at which they were collected, and the geographical area. This could include genetic differences as well as chemical differences and even the sex of the beetle and the temperature/humidity at which the beetle pupated could be a factor [29]. This would be analogous to cholesteric liquid crystals whose pitch is temperature dependent [30]. Other factors after death are less likely to affect the reflection spectra as much in terms of shape. Such factors include the storage conditions, such as humidity and temperature, the manner in which the beetle was prepared, UV exposure as well as the length of storage.

## 7. Conclusion

The study of beetles which selectively reflect LCP light goes back over 100 years, with many different areas of the scientific community contributing to the field. The variation of LCP reflection spectra within and between species of *Lomaptera* beetles was investigated. The LCP reflection spectra were classified into six shape types and also by pitch values. Some species spectra (e.g. *L. soror*) varied more than others (e.g. *L. helleriana*). A wide range of colours were seen, from blue to red and even into the infrared, although the vast majority of beetles were green. From the analysis of the spectra, it appears that classification by this method alone cannot fully distinguish between species due to variation within the species and also similarities between them. This study is based upon biological samples and as such there are many unknown variables which occur, these include the beetle's development and the chemical deficiencies, and how these will alter the optical properties is not fully understood. It can in some cases be seen that there is a clear difference between certain species in their range of spectral shapes, in that a small variation in structure results in the change in spectral shape.

## Supplementary Material

Details of Simulations and Parameter Variation
