# Supplementary material for: Variation in the circularly polarized light reflection of Lomaptera (Scarabaeidae) beetles
Source: J R Soc Interface. 2016 Jul;13(120):20160015. doi: 10.1098/rsif.2016.0015 (PMC4971215; doi:10.1098/rsif.2016.0015)
Supplement: Details of Simulations and Parameter Variation [file rsif20160015supp1.doc]

**Supplementary Material: Details of Simulations and Parameter Variations**

**S.1 Description of Modelling Technique**

The modelling of the observed spectra was carried out using functions from the BTFToolbox freeware published in association with the paper’s Ref. [22]. The Toolbox can be downloaded from <http://www.worldscientific.com/r/P962-supp>.

This section gives a brief overview of how the reflection spectra are calculated using Toolbox functions.

Fig. S1: Schema and notation used in modelling propagation through a birefringent stack.

Propagation is presumed to be along a principal axis of a birefringent medium in which the transverse (complex) principal indices
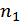
 and
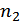
 are specified (see Fig. S1). Each chiral stack consists of
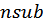
 birefringent layers in which the principal axes of each layer are successively rotated by a fixed increment. The increment is determined as
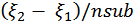
, where
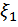
 and
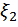
specify the orientation of the principal axes of the first and last layers of the stack respectively.

The wavelength is specified in units of a given Bragg wavelength using
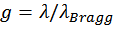
.

A call to the Toolbox function hmat via


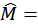
hmat(
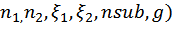


returns the so-called characteristic matrix (
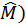
, relating the transverse electromagnetic fields at the plane
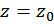
 to the transverse fields at the plane
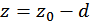
, where
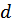
 is the thickness of the stack.

Most of our simulations explore
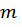
stacks in series, for which the resultant characteristic matrix is calculated as


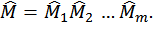


The circular reflection spectra
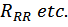
 are then calculated via a call to the Toolbox function reflect:


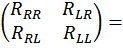
 reflect
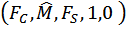
 ,

where
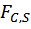
 are matrices accounting for the substrate and cover of the stack (in our case presumed to be index matched isotropic media), and the last two arguments specify the polarization basis as circular.

**S.2 Parameters used and varied in simulations**

**S.2.1**

Given the size of parameter space available it is possible that, for a given structural form, several different parameter sets yield very similar spectra. Thus we do not claim that we have achieved a detailed quantitative agreement between the chiral parameters used in the model, and those present in a given sample. However, as the following examples illustrate, models associated with different structural forms yield qualitatively distinct spectra. A Bragg reflection peak punctuated with a central transmission dip for example, might be due to either a twist defect or two gratings in series with slightly differing pitches. Below we show that the spectra for these two cases are very different, allowing the structural form to be uniquely inferred (cf. Fig. 5 in the paper). We define figures of merit for the two types of double peaked spectra, whereby the result of deviations from the optimized parameter values reported in the main paper can be quantified.

**S.2.2 Parameter Ranges Used in Simulations:**

Mean refractive index,
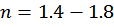
 cf. [1], Fig. 6 and Table 1 therein. The introduction of [2] also gives a range of 14.-1.8.

Extinction coefficient
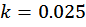
. cf. [1], Fig. 6 and Table 1 therein. Can vary depending on sample contamination.

Chitin layer thickness,
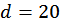
nm cf. [3].

Birefringence,
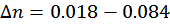
 cf. [4], p.13.

Number of half-turns per stack, ~10 This is typical as determined from SEM micrographs.

**S.2.3 Notation**

In reporting the simulations we use the following notation


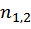
 Complex principal refractive indices.


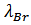
 Bragg wavelength


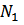
 Number of half-turns in first stack


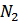
 Number of half-turns in second stack


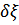
 Angular discontinuity

**S.3 Discussion of Paper’s Fig. 5: Two Closely Spaced Peaks**

We first define (see Fig. S1)


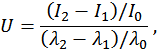


and then define a figure-of-merit for this spectral type as


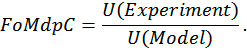


The closer
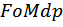
 is to one, the better the theoretical curve is judged to fit the experimental data.

Fig.S2: Parameters used to define
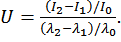


The structure we proposed to describe the experimentally observed spectrum in Fig. 5 of the paper is a discontinuity in the orientation of the birefringent axes (a ‘twist defect’), a context well-known to produce a dip in the reflection spectrum within the Bragg zone [5]. This consists of an abrupt change (
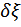
 ) in the orientation of the principal axes in the transverse plane. The location of the angular discontinuity is defined by the number of half-twists before (
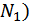
 and after (
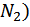
 the defect. The best fit (
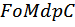
 =0.92) we obtained is shown in Fig. S3, which reproduces Fig. 5 of the paper. Note that the discontinuity is slightly offset from
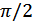
 (the value known to have the most pronounced effect [5]), and the defect location is slightly off-centre (
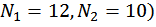
.

Figures S4 and S5 show the effect of varying the value of
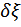
 away from
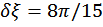
 (to
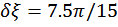
 and
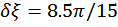
 respectively). It is seen that in both cases the calculated spectrum deviates from the optimized case, the deviation being quantified through the poorer
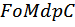
 values (
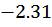
 and 0.39 respectively). Changing the location of the twist discontinuity (from
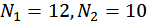
 to
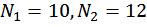
) also results in a reduction of
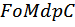
 to 0.6, as shown in Fig. S6.

In Fig. S7 we attempt to use a different structural model to explain the spectrum. Here, two chiral stacks are placed in series with distinct Bragg wavelengths (
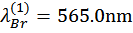
,
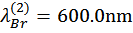
). It might be supposed that two closely spaced Bragg peaks could reproduce the single peak with a dip as observed experimentally. However, we have found that over the parameter ranges we have tested, the merged spectrum is radically distinct from the observed spectrum. As the peaks merge, the sidebands of one peak appears to contaminate the Bragg peak of the other, typically resulting in highly asymmetric heights for
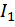
and
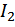
. Adjusting the number of layers in each stack does not appear to improve the fit. For the example shown in Fig. S7,
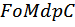
 = -2.5
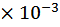
. It would appear that the spectral type we observed in Fig. 5 is better explained as being the result of a twist discontinuity in a single Bragg stack.

Fig. S3: Twist defect. Model parameters:
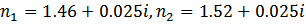
,
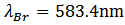
,
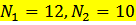
,
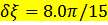
. *FoMdpC* = 0.92.

Fig. S4: Twist defect. Model parameters:
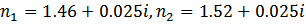
,
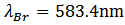
,
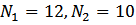
,
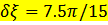
. *FoMdpC* = -2.31.

Fig. S5: Twist defect. Model parameters:
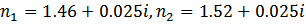
,
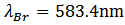
,
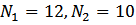
,
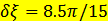
. *FoMdpC* =0.39.

Fig. S6: Twist defect. Model parameters:
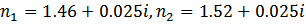
,
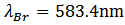
,
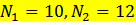
,
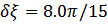
. *FoMdpC* = 0.60.

Fig. S7: Double Bragg structure: Model parameters:
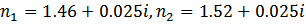
,
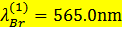
,
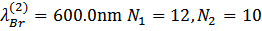
, *FoMdp* = -2.5
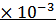
.

**S.4 Discussion of Paper’s Fig. 6: Two distinct peaks**

When the two peaks are far apart (i.e. separated by more than the Bragg bandwidth of either peak), then two separate Bragg gratings are present. In this case we define (see Fig. S8)


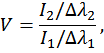


and then a figure of merit associated with this spectral type according to


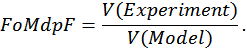


Deviations of
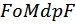
 from unity for a given model signify a poorer fit to the experimental data. Fig. S9 reproduces Fig. 6 of the paper, showing an optimized model fit yielding a value of
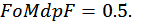
For these simulations we note that the absorption is significantly weaker than for the case considered in the previous section. We found that even modest absorption almost eliminated the longer wavelength Bragg peak entirely. The principal challenge in achieving a good fit is to balance the heights of the two peaks whilst maintaining the correct spectral bandwidths. This can be achieved by varying the number of half turns in each stack, as this determines the peak height without altering the bandwidth. The effect of deviating from the optimized values of
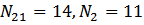
 is shown in Fig. S10 which yields *FoMdpfF*=6.6.

Fig.S8: Parameters used to define
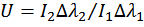


Fig. S9: Double Bragg structure. Model parameters:
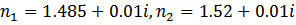
,
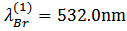
,
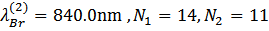
. *FoMdpfF*=2.2.

Fig. S10: Double Bragg structure. Model parameters:
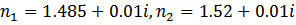
,
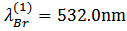
,
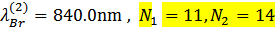
. *FoMdpfF*=6.4.

**S.5 Spectra with oscillations**

Fig. S11 reproduces Fig.9 from the paper, wherein the presumed model was one with two twist discontinuities present in a Bragg grating of unique pitch. With optimized angular discontinuities (
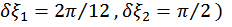
defect locations (
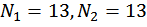
,
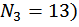
 the observed oscillations on the longer wavelength side of the Bragg wavelength are reproduced. However, the experimental spectrum shows regular oscillations occurring well beyond the Bragg zone, which are not reproduced in the double-twist-discontinuity model. Our searches for an alternative structure reproducing this feature were inconclusive.

Fig. S11: Double Twist defect. Model parameters:
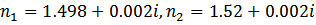
,
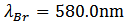
,
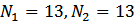
,
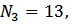

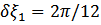
,
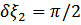
.

References

[1] Azofeifa, D. E., Arguedas, H. J. and Vargas, W. E., ‘Optical properties of chitin and chitosan biopolymers with application to structural color analysis’, *Optical Materials*, **35**, 175-183, (2012).

[2] Arwin, H., Berlind, T., Johs, B. and Järrendahl, K., ‘Cuticle structure of the scarab beetle *Cetonia aurata* analysed by regression analysis of Mueller-matrix ellipsometric data’, *Optics Express*, **21**, 22645-22656, (2013).

[3] Nikolov, S., Fabritius, H., Petrov, M., Friák, M., Lymperiakis, L., Sachs, C., Raabe, D. and Neugebauer, J., ‘Robustness and optimal use of design principles of arthropod exoskeletons studied by ab initio-based multiscale simulations’, *Journal of the Mechanical Behavior of Biomedical Materials*, **4**, 129-145, (2011).

[4] Wu, X., ‘*Structure-property-relations of Cuticular Photonic Crystals Evolved by Different Beetle Groups (Insecta, Coleoptera)*’, epubli GmbH, Berlin (2014).

[5] Hodgkinson,I.J., Wu,Qi-H., De Silva,L., Arnold,M., McCall,M.W., Lakhtakia,A., 'Supermodes of Chiral Photonic Filters with Combined Twist and Layer Defects', *Physical Review Letters*, **91**, 223903, (2003).
